# Nutritional bioactives for preventing recurrent urinary tract infections in children: microbiome-mediated mechanisms and clinical implications

**DOI:** 10.3389/fnut.2026.1855301

**Published:** 2026-06-10

**Authors:** John Dotis

**Affiliations:** Third Department of Pediatrics, Hippokration Hospital, Aristotle University of Thessaloniki, Thessaloniki, Greece

**Keywords:** antimicrobial stewardship, d-mannose, gut–bladder axis, non-antibiotic prevention, proanthocyanidins

## Abstract

Recurrent urinary tract infections in children represent a common clinical challenge associated with repeated antibiotic exposure and rising antimicrobial resistance. These limitations have intensified interest in non-antibiotic preventive strategies, particularly nutritional bioactives capable of modulating host–microbe interactions. This review provides a clinically oriented synthesis of current evidence on dietary and nutraceutical interventions for the prevention of recurrent pediatric urinary tract infections. Particular attention is given to the limited availability of high-quality pediatric-specific evidence and the heterogeneity of current clinical data. It focuses on cranberry-derived type A proanthocyanidins, probiotics, selected micronutrients and D-mannose. Key mechanistic pathways are also highlighted, including inhibition of uropathogen adhesion, microbiome-mediated biotransformation of bioactives into anti-inflammatory metabolites, and modulation of host immune and epithelial responses within the gut–bladder axis. Available evidence suggests that cranberry products standardized to deliver approximately 36 mg/day of type A proanthocyanidins may reduce recurrence risk, whereas probiotics and vitamins A, C, and D may provide adjunctive benefits through microbiome modulation and enhancement of innate immune responses. However, substantial heterogeneity in study design, variability in formulations and dosing, and the limited availability of high-quality pediatric randomized trials remain important limitations. Building on current evidence, we propose a pragmatic multimodal framework for non-antibiotic prevention in children that integrates nutritional strategies with clinical risk stratification and individualized care, with particular attention to bioavailability, dose standardization and pediatric-specific factors such as age-related microbiome maturation. Future research should prioritize biomarker-driven endpoints, microbiome-informed stratification and adequately powered pediatric studies to define responders and optimize personalized prevention strategies.

## 1 Introduction

Urinary tract infections (UTIs) remain among the most common bacterial infections in childhood and represent a substantial source of antimicrobial exposure. In pediatric populations, recurrent urinary tract infections (rUTIs), typically defined as two or more episodes within 6 months or three within 1 year, are associated not only with repeated clinical encounters but also with cumulative antibiotic use and increasing selective pressure for antimicrobial resistance (AMR) (1, 2). Importantly, recurrent infections frequently occur in children with coexisting functional or structural urinary tract abnormalities, including bladder bowel dysfunction and vesicoureteral reflux, further contributing to recurrence susceptibility and clinical complexity. Thus, rUTIs may be considered a clinically relevant contributor to the broader AMR challenge. Recurrent infections are additionally linked to increased healthcare utilization during early life, further emphasizing their broader clinical and public health relevance.

The rise of AMR has intensified the need for strategies that reduce unnecessary antibiotic exposure, particularly in early life, where antimicrobial use may influence both resistance patterns and microbiome development (3, 4). In this context, long-term antibiotic prophylaxis, although still used in selected cases, provides modest benefit and raises concerns regarding resistance and microbiome disruption and long-term ecological effects (5, 6). These limitations have driven increasing interest in non-antibiotic preventive approaches.

Cranberry-derived products, probiotics, micronutrients and D-mannose have been investigated as potential alternatives. Recent meta-analyses support a possible role for cranberry products, particularly in relation to adequate proanthocyanidin (PAC) dosing and treatment duration (7, 8). Nevertheless, important limitations remain, including the relative scarcity of pediatric-specific data, heterogeneity among formulations and study protocols, and the predominance of single-intervention approaches despite the multifactorial pathophysiology of rUTIs.

From a nutritional and mechanistic perspective, these interventions may act through interconnected microbiome-mediated and host-response mechanisms rather than isolated effects. Cranberry PACs, for example, are extensively metabolized by the gut microbiota into bioactive compounds that may influence bacterial adhesion and urothelial signaling epithelial responses (9), while probiotics and micronutrients may contribute to modulation of microbial composition and immune function. Collectively, these observations suggest that effective prevention strategies may require integration of multiple biological pathways within the broader hypothesized gut–bladder axis. Available evidence derives from geographically heterogeneous pediatric and adult cohorts, although important regional variability in dietary patterns, microbiome composition, healthcare practices and formulation availability should be acknowledged.

The aim of the present review is to provide a clinically oriented and mechanistically informed synthesis of non-antibiotic strategies for preventing rUTIs in children, with particular emphasis on microbiome-mediated mechanisms, host–microbe interactions and their potential implications within antimicrobial stewardship frameworks. Summarizing current evidence, this review also attempts to integrate emerging mechanistic and translational concepts that may help inform future individualized preventive approaches in pediatric clinical practice.

## 2 Methods

This narrative review was based on a structured literature search designed to identify clinically relevant and mechanistically informative evidence regarding non-antibiotic nutritional strategies for the prevention of rUTIs in children. PubMed/MEDLINE, Scopus, and Web of Science were searched for studies published up to May 2026. Search terms included combinations of “recurrent urinary tract infection,” “children,” “pediatric,” “cranberry,” “proanthocyanidins,” “probiotics,” “vitamin C,” “vitamin D,” “microbiome,” “gut–bladder axis,” “urobiome,” “D-mannose,” “non-antibiotic prevention,” “antimicrobial stewardship,” and “urinary tract infection prophylaxis”.

Priority was given to pediatric-focused clinical studies, randomized controlled trials, systematic reviews, meta-analyses, and guideline-relevant evidence. When pediatric-specific data were limited, selected adult translational studies and experimental mechanistic investigations were included to support biological plausibility and mechanistic interpretation, although the limitations of extrapolating these findings directly to pediatric populations were carefully acknowledged.

Studies were selected according to their relevance to rUTI prevention, mechanistic contribution, pediatric applicability, and clinical interpretability. Evidence synthesis was performed narratively rather than systematically because of substantial heterogeneity across available studies regarding populations, intervention formulations, dosing regimens, treatment duration, microbiological endpoints, and definitions of recurrence.

Accordingly, the objective of this review was not to generate pooled quantitative estimates, but rather to integrate current pediatric clinical evidence with emerging mechanistic and translational concepts that may help inform a clinically oriented framework for individualized non-antibiotic prevention strategies in children with rUTIs.

## 3 Biological basis of non-antibiotic nutritional prevention of recurrent urinary tract infections

### 3.1 Cranberry-derived proanthocyanidins and anti-adhesion mechanisms

Cranberry products exert their primary effect through PACs, particularly A-type PACs, which inhibit the adhesion of uropathogenic *Escherichia coli* to uroepithelial cells. This process is mediated by interference with the FimH adhesin located on type 1 pili, thereby preventing bacterial attachment to mannose-containing receptors on the urothelial surface (10, 11). By targeting this early and critical step in infection pathogenesis, cranberry-derived compounds may reduce colonization without exerting direct bactericidal pressure, a feature that is particularly relevant in the context of AMR.

In addition to type 1 fimbriae, experimental evidence suggests that cranberry constituents may also affect P-fimbrial adhesion and biofilm formation, further limiting bacterial persistence within the urinary tract (12). Importantly, this anti-adhesion mechanism is non-lethal to bacteria and therefore unlikely to promote resistance selection, distinguishing it from conventional antimicrobial strategies.

### 3.2 Microbiome-mediated metabolism and the gut–bladder axis

From a nutritional perspective, the biological activity of cranberry PACs is closely linked to their interaction with the gut microbiota. Due to their high molecular weight and limited absorption in the small intestine, a substantial proportion of PACs reaches the colon intact, where they undergo extensive microbial biotransformation into lower molecular weight phenolic metabolites (9).

These metabolites, including phenylacetic and phenylpropionic acid derivatives, may enter systemic circulation and contribute to host biological effects, including potential modulation of inflammatory pathways and epithelial function (9, 13). Emerging evidence suggests that these microbiome-derived metabolites may exert systemic immunomodulatory effects beyond the urinary tract, including possible regulation of inflammatory signaling pathways and host defense responses. Such effects may involve downstream modulation of cytokine expression and epithelial defense mechanisms, potentially extending beyond local urothelial interactions (13).

This microbiome-dependent metabolism introduces an additional layer of inter-individual variability, as the composition and functional capacity of the gut microbiota may influence the magnitude and nature of the host response. These processes support the hypothesis of a broader gut–systemic–urinary axis, rather than a purely local anti-adhesion mechanism, providing a more integrated framework for understanding the biological effects of cranberry-derived compounds (9, 13).

Moreover, cranberry-derived compounds have been shown to alter gut microbial composition, promoting the growth of beneficial taxa such as *Lactobacillus* and *Bifidobacterium*, while potentially suppressing pathogenic species (9). Such shifts toward a more favourable microbial profile may potentially influence susceptibility to UTIs through gut–urinary axis interactions, although this concept remains incompletely defined in pediatric populations.

### 3.3 Host epithelial and immune responses

Beyond microbial interactions, non-antibiotic strategies may influence host defense mechanisms at the level of the urothelium. Cranberry-derived metabolites have been associated with reduced bacterial-induced inflammation, possibly through experimental and translational modulation of signalling pathways involved in innate immunity, including Toll-like receptor (TLR)-mediated responses and downstream cytokine production (13). These findings raise the possibility that cranberry compounds may not only prevent bacterial adhesion but also attenuate the host inflammatory response to infection.

Micronutrients such as vitamins A, C and D further contribute to host defence by enhancing epithelial barrier integrity and promoting the expression of antimicrobial peptides, including cathelicidin and defensins (14). Similarly, probiotics may exert indirect effects by restoring microbial balance and reducing pathogen colonization, although their clinical impact remains variable and strain-dependent (15).

### 3.4 Toward an integrated biological model

Taken together, these findings support a model in which non-antibiotic preventive strategies act through complementary and overlapping mechanisms, including anti-adhesion effects, microbiome modulation and host immune regulation. This integrated framework may better reflect the multifactorial nature of rUTIs compared with single-target approaches.

**Figure 1:**
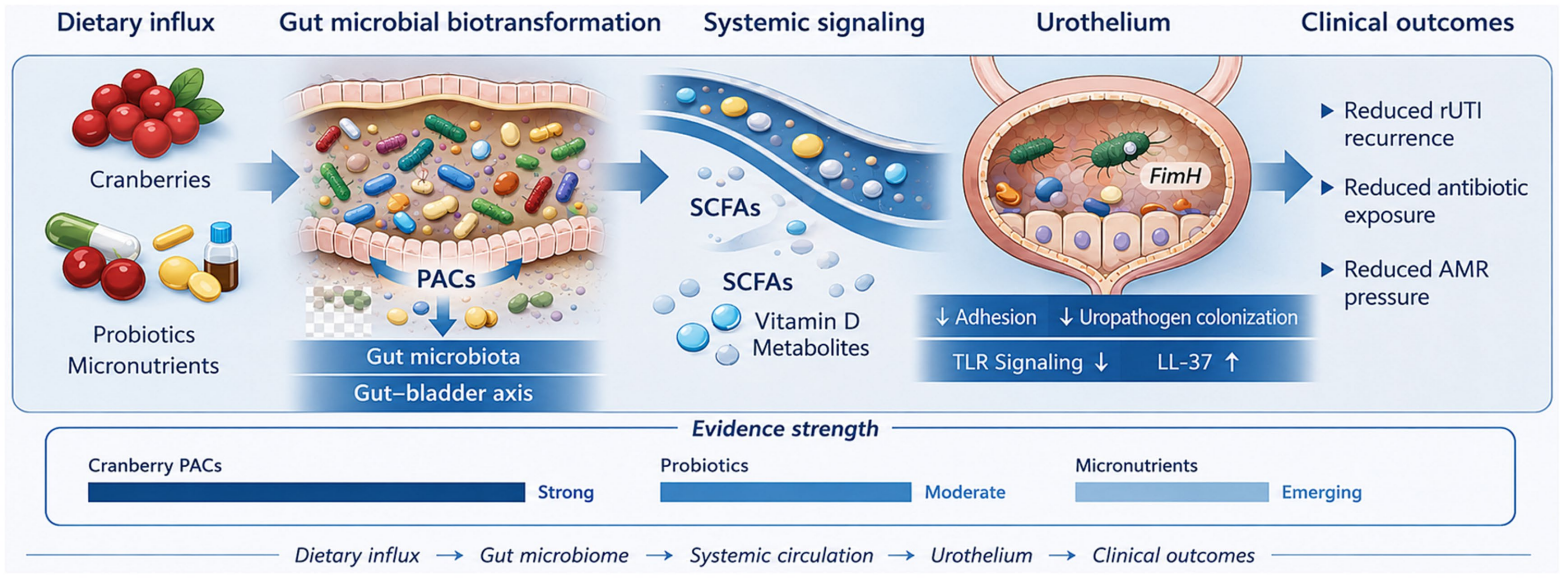
Nutrition- and microbiome-mediated mechanisms underlying non-antibiotic prevention strategies for recurrent urinary tract infections in children. The schematic illustrates integrated pathways through which PACs, probiotics, and micronutrients may contribute to rUTI prevention via microbiome-mediated biotransformation, host immune modulation, reduced bacterial adhesion, and altered urothelial signaling within the gut–bladder axis. The proposed mechanisms are based on experimental, translational, and clinical evidence and should be interpreted as interconnected biological processes rather than isolated effects. Source: Author’s original work. PACs, proanthocyanidins; SCFAs, short-chain fatty acids; TLR, Toll-like receptor; LL-37, cathelicidin antimicrobial peptide; AMR, antimicrobial resistance; rUTIs, recurrent urinary tract infections. Figure was generated using ChatGPT and the prompt used can be found in Supplementary Table 1.

Importantly, such mechanisms are consistent with a preventive paradigm that minimizes selective pressure for AMR while maintaining biological effectiveness. However, despite increasing mechanistic insight, the translation of these pathways into consistent clinical benefit remains dependent on factors such as dosing, formulation, treatment duration and host-specific characteristics. Further pediatric-focused studies are required to clarify these interactions and optimize their clinical application. The principal nutrition- and microbiome-mediated mechanisms underlying these strategies are illustrated in Figure 1.

## 4 Current evidence landscape

### 4.1 Burden of rUTIs in children

UTIs are among the most frequently encountered bacterial infections in childhood and continue to represent a notable source of morbidity, healthcare use and antibiotic exposure (1, 16). rUTIs affect a considerable subset of pediatric patients and are linked to repeated medical consultations, caregiver concern and in selected cases, potential renal parenchymal damage (2). In addition, recurrent infections contribute to repeated healthcare interactions and cumulative antimicrobial exposure, particularly during early childhood (1). Beyond individual morbidity, recurrent infections contribute to cumulative antibiotic exposure and repeated healthcare interactions, underscoring their broader clinical and public health relevance.

### 4.2 Limitations of antibiotic prophylaxis

For many years, long term antibiotic prophylaxis has been routinely implemented in children considered at increased risk of recurrence. However, growing evidence indicates that its clinical benefit may be limited and this must be balanced against well recognized disadvantages, including adverse drug effects as well as the development of AMR (5). Even in carefully selected groups, the reduction in recurrence appears modest, while the broader ecological consequences of prolonged antibiotic exposure are an increasing concern (6). In addition, prolonged prophylaxis may alter the microbiological profile of subsequent infections, potentially leading to more resistant or difficult to treat pathogens. These challenges have stimulated interest in alternative, non-antibiotic strategies aimed at preventing recurrence while reducing unintended harms.

### 4.3 Mergence of non-antibiotic strategies

A variety of non-antibiotic interventions has been explored, including cranberry derived products, probiotics, micronutrient supplementation and more recently, D mannose. Despite their theoretical rationale and widespread clinical application, the supporting evidence remains variable and often inconclusive. Probiotics, for instance, have been proposed to help reestablish a balanced urogenital microbiota, yet current data do not consistently demonstrate a significant clinical reduction in recurrence rates (17). Likewise, micronutrients such as vitamins A, C and D have been investigated as adjunctive options due to their potential immunomodulatory effects, but strong pediatric evidence remains scarce (14). D mannose, which may prevent bacterial adherence to the urothelial surface, represents another promising approach. However, available data are limited and characterized by low certainty (18). Overall, while these approaches are increasingly incorporated into clinical practice, their relative efficacy and optimal role remain unclear.

### 4.4 Fragmentation of current evidence

In contrast, cranberry products have been more extensively investigated than other non-antibiotic options. Their proposed mechanism of action, largely attributed to PACs that impair bacterial adhesion to uroepithelial cells, is supported by both experimental and clinical findings (10). Recent studies suggest a possible reduction in UTI recurrence in certain populations, including children; however, variability in formulations, dosing regimens and study methodologies complicates interpretation, thereby limiting broader applicability (7). Importantly, there is no clear agreement regarding optimal dosing or patient selection and comparative effectiveness relative to other non-antibiotic strategies remains insufficiently established. Taken together, the existing literature reveals a key inconsistency: although multiple non-antibiotic strategies are available and commonly used in practice, the supporting evidence is fragmented, sometimes conflicting and rarely focused specifically on pediatric populations (2, 16). Furthermore, most studies have assessed individual interventions in isolation, without considering whether a combined, multi target strategy, integrating anti adhesion effects, microbiome modulation and immune support, may be necessary to achieve meaningful clinical benefit. Importantly, many of these proposed interactions are primarily supported by mechanistic, experimental, or translational evidence rather than clinically validated pediatric outcome data. This lack of integrative evidence represents a critical gap between research findings and real-world clinical decision making.

### 4.5 Aim of the review

Within this framework, the objective of the present review is to deliver a clinically focused synthesis of non-antibiotic strategies for preventing rUTIs in children. By integrating contemporary evidence with clinically relevant observations, this review aims to connect research findings with real-world pediatric practice, emphasizing both current evidence signals and existing limitations, while also identifying priorities for future investigation. Emphasis is placed on translating heterogeneous evidence into practical considerations for clinicians managing children with recurrent infections.

## 5 Non-antibiotic strategies evidence synthesis

### 5.1 Cranberry

Cranberry-based interventions are among the most studied non-antibiotic strategies for preventing rUTIs. Consistent with earlier observations, their effect is primarily attributed to A-type PACs, which reduce bacterial attachment by interfering with FimH-dependent adhesion of uropathogenic *E. coli* to the urothelium (11, 19). By targeting this early step, cranberry compounds may limit colonization. They also inhibit hemagglutination and biofilm formation (11), without exerting bactericidal activity, thereby potentially minimizing AMR selection.

Recent high-quality systematic reviews and meta-analyses have reinforced the clinical relevance of cranberry products. A large-scale synthesis incorporating approximately 50 randomized controlled trials and more than 8,000 individuals indicates that cranberry consumption is associated with a lower incidence of symptomatic, culture-verified UTIs, with particularly consistent effects observed in pediatric population (7). In children, the relative risk reduction has been estimated at nearly half (RR ≈ 0.46), making cranberry products among the non-antibiotic strategies with the most consistent pediatric evidence currently available. Additional pediatric-focused analyses have reported similar findings, demonstrating decreased likelihood of symptomatic infection episodes and recurrence burden during periods of cranberry use (20). Taken together, these data provide one of the most consistent clinical signals among currently available non-antibiotic strategies.

An emerging theme in the literature is the importance of adequate PAC intake for achieving clinical benefit. Meta-analytic evidence suggests that formulations delivering at least 36 mg of PACs per day are linked to meaningful reductions in UTI risk, whereas lower amounts appear insufficient to produce significant effects (8). Available data also indicate that sustained administration, typically for at least 3–6 months, is required to achieve clinically meaningful reductions in recurrence, supporting both dose-dependent and duration-dependent prophylactic effects (7, 8). These findings indicate that adequate PAC exposure (≥36 mg daily) represents a critical threshold for efficacy rather than an optional dosing consideration, and that lower doses are unlikely to confer clinically meaningful protection.

In addition to their anti-adhesive properties, cranberry constituents may exert broader biological effects that contribute to their protective role. These include potential modulation of intestinal microbiota, production of active metabolites, and interference with both biofilm formation and inflammatory signalling pathways (13, 19). Emerging evidence further suggests that microbiome-mediated biotransformation of cranberry polyphenols may generate bioactive metabolites capable of exerting systemic immunomodulatory effects, although direct pediatric clinical evidence supporting these mechanisms remains limited. Collectively, these observations are consistent with the hypothesis of a broader gut–systemic–urinary axis rather than a purely local mechanism of action. The diverse chemical composition of cranberry products, encompassing polyphenols, flavonoids and phenolic acids, may partly explain variability in effectiveness across formulations and underscores the importance of product-specific characteristics.

Despite these promising observations, several limitations should be acknowledged when interpreting the current evidence. Considerable heterogeneity exists among studies in terms of cranberry formulation, PAC concentration, dosing strategies, treatment duration and characteristics of the study populations, which complicates direct comparisons and limits generalizability (7, 8). A key challenge is the lack of standardized methods for quantifying PAC content across products, making it difficult to clearly define dose–response relationships and potentially contributing to inconsistent clinical findings. Additional complexity arises from substantial variability among commercially available cranberry formulations, including juices, concentrated extracts, capsules, and combination products. Juice-based preparations may contain lower or inconsistent PAC concentrations and frequently include added sugars, whereas standardized extract or capsule formulations may allow more reliable delivery of target PAC doses. Moreover, differences in PAC quantification methods, particularly the use of DMAC versus non-standardized analytical approaches, further complicated comparison across studies and interpretation of clinical efficacy (8, 19). Although evidence in children is growing, the number of rigorously designed pediatric trials remains relatively small. In addition, important questions remain regarding optimal dosing regimens, duration of prophylaxis and effectiveness in specific subgroups such as those with underlying urinary tract abnormalities (7, 20).

Overall, cranberry products appear to represent a viable non-antibiotic strategy for reducing the risk of rUTIs, particularly in otherwise healthy pediatric populations. However, their effectiveness seems to depend on achieving sufficient PAC exposure, selecting appropriate formulations and tailoring use to individual patient characteristics. Consequently, cranberry supplementation should be considered as part of a broader, individualized prevention strategy rather than a universally effective solution. Further well-designed pediatric trials are warranted to refine dosing strategies and better define patient selection criteria. Collectively, these findings support the concept that non-antibiotic prevention of rUTIs may depend not only on direct anti-adhesive mechanisms, but also on potential modulation of host–microbiome interactions.

### 5.2 Probiotics

Probiotics have been explored as a potential non-antibiotic approach for preventing rUTIs, largely due to their ability to influence the composition of the urogenital microbiome and reduce colonization by pathogenic organisms. *Lactobacillus* strains are believed to provide protective effects through several mechanisms, such as outcompeting harmful bacteria, producing antimicrobial compounds and supporting the integrity of the mucosal barrier. These mechanisms are well described in pediatric-focused reviews and support the biological plausibility of probiotics in infection prevention (15, 21). Accordingly, probiotic supplementation has gained increasing attention as a potential adjunct or alternative to antibiotic prophylaxis (22).

Despite this strong theoretical basis, clinical data evaluating probiotics for rUTI prevention remain heterogeneous and inconclusive. Earlier systematic reviews and meta-analyses generally failed to demonstrate a statistically significant reduction in infection rates or recurrence when probiotics were administered alone, with pooled analyses generally demonstrating either modest or statistically non-significant reductions in recurrence rates compared with placebo (17, 23). More recent evidence focusing on pediatric cohorts indicates a possible but limited benefit, with pooled effect estimates remaining close to the null effect, thereby suggesting a limited and clinically variable effect (21). Importantly, considerable variability exists across studies regarding probiotic strains, dosing regimens, treatment duration and baseline patient characteristics, which further complicates interpretation of efficacy. Collectively, these findings underscore ongoing uncertainty regarding both the magnitude and clinical importance of probiotic efficacy.

Evidence from randomized controlled trials further reflects this inconsistency. For example, a recent double-blind, placebo-controlled trial investigating *Lactobacillus rhamnosus* PL1 and *Lactobacillus plantarum* PM1 in children with rUTIs reported a decrease in infection frequency over time in the probiotic group; however, comparisons between the probiotic and placebo groups did not achieve statistical significance (24) This recurring pattern has also been observed in other pediatric probiotic studies and may reflect non-specific effects, including closer follow-up, improved adherence to preventive measures, or increased healthcare engagement.

There is some indication that probiotics may offer greater value when used alongside other interventions rather than independently. Earlier meta-analyses suggest that combining probiotics with antibiotic prophylaxis may lead to modest reductions in recurrence rates compared with antibiotics alone, although the practical significance of this effect remains uncertain (23). Similarly, broader evaluations of non-antibiotic preventive measures in pediatric populations report low- to moderate-certainty evidence supporting a possible benefit of probiotics relative to placebo (21, 22). Importantly, probiotics are generally well tolerated and do not contribute to AMR, representing a notable advantage in long-term prevention strategies (17).

In summary, current evidence does not support probiotics as a reliably effective standalone therapy for preventing rUTIs in children. Although their use is biologically plausible and associated with a favorable safety profile, their clinical impact appears modest and variable, with potential influence from factors such as strain specificity, dosage and individual patient characteristics (21, 24). Consequently, probiotics are more appropriately considered as part of a multifaceted preventive strategy rather than a primary substitute for established preventive approaches.

### 5.3 Micronutrients – vitamins

Micronutrient status has emerged as a modifiable factor influencing susceptibility to UTIs, with increasing interest in the role of vitamins as adjunctive or preventive strategies. Among these, vitamins A, C and D have been most extensively investigated owing to their immunomodulatory, antioxidant and epithelial protective properties. Narrative syntheses support a potential role of vitamin supplementation in modulating infection risk, although clinical evidence remains variable (14, 25). Mechanistic and clinical data further suggest that micronutrient deficiencies may impair host defense and increase susceptibility to recurrent infections (14, 26).

Vitamin D has received particular attention in recent years. Observational studies consistently demonstrate an association between low serum 25 hydroxyvitamin D levels and increased risk of UTIs in pediatric populations, with lower concentrations reported in affected children compared with healthy controls (26, 27). This association is biologically plausible, as vitamin D enhances innate immune responses through induction of antimicrobial peptides such as cathelicidin and defensins, while also supporting epithelial barrier integrity and potentially modulating inflammatory signalling (14, 16). In addition, vitamin D deficiency has been linked to more severe clinical presentations, including pyelonephritis, suggesting a possible role in both susceptibility and disease severity (27). However, despite consistent observational signals, interventional evidence remains limited and whether supplementation reduces recurrence risk is unclear (2, 14). Currently available evidence is derived predominantly from observational and case–control studies, whereas adequately powered pediatric interventional trials remain scarce.

Beyond vitamin D, combined micronutrient deficiencies may further contribute to infection risk. Case control data indicate that children with UTIs frequently exhibit concurrent deficiencies in vitamin D and trace elements such as zinc, supporting the concept of broader immune impairment rather than isolated deficiency (26). These findings align with the broader understanding that host related factors, including immune maturation and nutritional status, play a central role in susceptibility to recurrent infections in childhood (16).

Vitamin A represents another micronutrient with potential relevance in UTIs. Its role in maintaining epithelial integrity and regulating mucosal immunity provides a strong mechanistic rationale, particularly in the context of ascending infections. Clinical evidence, although limited, suggests that vitamin A supplementation may improve clinical symptoms and reduce renal parenchymal injury in children with acute pyelonephritis when used alongside standard antimicrobial therapy (14, 28). Randomized pediatric data have also suggested a potential reduction in renal scarring following adjunctive vitamin A supplementation in acute pyelonephritis (28). These effects are likely mediated through enhanced epithelial repair and possible modulation of inflammatory pathways central to renal injury following infection (16).

Vitamin C has also been proposed as a supportive intervention due to its antioxidant activity and potential to influence the urinary environment. Experimental data suggest that ascorbic acid may exert indirect antimicrobial effects through modulation of oxidative and nitrosative pathways, while clinical observations indicate a possible role in symptom reduction when used as adjunctive therapy (14). However, evidence remains limited and inconsistent, particularly in pediatric populations and its role in preventing recurrence remains unclear.

Taken together, current evidence suggests that micronutrients may play a contributory but not definitive role in the prevention and management of rUTIs. While associations between vitamin deficiencies and increased susceptibility are consistently reported, clinical trials evaluating supplementation strategies remain scarce and heterogeneous (14, 27). Importantly, in contrast to cranberry products, for which more consistent clinical signals have been reported, micronutrient-based interventions currently lack robust evidence from well-designed randomized trials (7, 20). The potential benefit of vitamin-based interventions likely depends on baseline nutritional status, individual risk factors and integration within broader preventive approaches. In clinical practice, targeted correction of documented deficiencies according to age-appropriate nutritional recommendations appears clinically justified, whereas routine supplementation in unselected populations cannot be recommended (14, 26).

### 5.4 D-mannose

D-mannose has been proposed as a non-antibiotic preventive strategy for rUTIs, based on its ability to interfere with bacterial adhesion to the urothelium. Specifically, D-mannose binds to the FimH adhesin located on type 1 pili of uropathogenic *E. coli*, thereby preventing attachment to uroepithelial cells and facilitating bacterial clearance through urination (11, 29). This anti-adhesion mechanism is biologically plausible and aligns with non-antibiotic strategies targeting early steps in infection pathogenesis, although its activity appears more narrowly targeted than the broader anti-adhesive profile described for cranberry-derived PACs (12).

Despite this strong mechanistic rationale, clinical evidence supporting the use of D-mannose remains limited and of low certainty. The most comprehensive Cochrane review to date identified only a small number of heterogeneous studies, with insufficient data to determine whether D-mannose is effective in preventing or treating UTIs and overall certainty of evidence rated as very low (18). Similarly, more recent meta-analytic data, primarily derived from adult populations, do not demonstrate a significant reduction in recurrence rates compared with control or antibiotic prophylaxis, highlighting uncertainty regarding its clinical utility (30). Considerable heterogeneity exists across available studies regarding patient selection, dosing regimens, treatment duration and comparator interventions, further limiting interpretation of efficacy.

Evidence in pediatric populations is particularly limited, and most available data are extrapolated from adult studies or small heterogeneous cohorts. Pediatric-specific randomized controlled trials remain largely unavailable. As a result, current evidence does not support the routine use of D-mannose as a standalone preventive strategy in children with rUTIs. While its favourable safety profile and biological plausibility make it an attractive candidate, clinical effectiveness remains uncertain (18, 31). Overall, D-mannose represents a mechanistically appealing but clinically insufficiently validated intervention. Its potential role, if any, is therefore more likely to be complementary rather than primary within multifaceted preventive strategies.

## 6 Clinical integration and future perspectives

### 6.1 Comparative interpretation of available strategies

The prevention of rUTIs in children remains a complex clinical challenge, requiring a careful balance between efficacy, safety, and long-term consequences of preventive strategies. Although antibiotic prophylaxis has historically represented the standard approach, concerns regarding modest clinical benefit, microbiome disruption, and the promotion of AMR have driven increasing interest in non-antibiotic alternatives (5, 6), with current guideline-oriented approaches supporting individualized, risk-adapted prevention strategies, particularly in children with recurrent or complicated infections (32).

Within this evolving framework, non-antibiotic interventions, including cranberry products, probiotics, micronutrients and D-mannose, demonstrate differing levels of mechanistic support and clinical evidence (16), with cranberry products consistently providing the most consistent evidence profile, particularly in pediatric populations. Data from randomized trials and meta-analyses suggest a clinically relevant reduction in recurrence risk, especially when adequate exposure to A-type PACs is achieved (7, 8, 20), further supported by earlier pediatric studies showing reductions in recurrence burden and antibiotic exposure (33, 34).

In contrast, probiotics and micronutrient-based strategies, although biologically plausible and generally well tolerated, are supported by more heterogeneous and less robust data (14, 22, 27), and therefore appear to have a primarily adjunctive role, with potential value in selected patients or as part of combined preventive approaches. Similarly, D-mannose represents a mechanistically attractive option; however, current evidence remains limited and of low certainty, particularly in pediatric populations (18, 30).

These differences highlight an important clinical principle: non-antibiotic strategies are not equivalent in terms of evidence strength and should therefore be applied within an individualized framework, integrating patient-specific factors such as age, recurrence pattern, underlying urological abnormalities, bowel dysfunction, prior antibiotic exposure, and family preferences into clinical decision-making (16). In otherwise healthy children with rUTIs, cranberry products may be considered a reasonable first-line non-antibiotic option, whereas other interventions are more appropriately used as adjunctive strategies. Importantly, several proposed microbiome-mediated and immunomodulatory mechanisms discussed throughout the present review are derived primarily from experimental, translational, or observational evidence, whereas clinically validated pediatric outcome data remain comparatively limited. A comparative summary of the principal non-antibiotic strategies, including their molecular mechanisms, evidence profile, and clinical applicability, is presented in Table 1.

**Table 1:** Mechanisms, evidence profile and pediatric considerations of non-antibiotic preventive strategies for recurrent urinary tract infections.

Intervention	Key bioactive compounds	Molecular mechanism of action	Certainty/level of evidence	Bioavailability/pharmacokinetics in children	Specific pediatric considerations	Clinical recommendation	Research gaps/future directions	Key references
Cranberry	Type-A PACs	Inhibition of FimH-mediated adhesion of uropathogenic *E. coli*; interference with P-fimbriae; potential modulation of host inflammatory pathways (including TLR-related signaling)	Moderate to High (strongest evidence among currently available non-antibiotic strategies, although pediatric-specific data remain comparatively limited)	Limited small intestinal absorption; gut microbiome-mediated metabolism to phenolic acids	Dose-dependent effect; standardized formulations (~36 mg PACs/day, measured by DMAC assay); adherence and formulation variability relevant	Preferred non-antibiotic option in selected pediatric populations (typically for 3–6 months)	Standardization of PAC measurement (DMAC vs. other assays); pediatric dose optimization; identification of responder subgroups	(7, 8, 20)
Probiotics	*Lactobacillus* spp. (e.g., *L. rhamnosus*, *L. plantarum*)	Modulation of microbiota; pathogen displacement; production of SCFAs (short-chain fatty acids); support of the hypothesized gut–bladder axis signaling and mucosal immunity	Low to moderate (heterogeneous RCTs and meta-analyses)	Strain-dependent colonization; transient microbiome modulation influenced by baseline composition	Strain-specific efficacy; potential benefit in children with gut dysbiosis or constipation; evidence derived from geographically heterogeneous cohorts	Adjunctive approach; may be considered in selected cases	Identification of optimal strains; role in multimodal strategies; microbiome-driven personalization	(21, 23, 24)
D-mannose	D-mannose	Competitive binding to FimH adhesins on type 1 fimbriae; inhibition of bacterial adhesion	Very low (limited and heterogeneous data; lack of pediatric RCTs)	Rapid absorption and urinary excretion; minimal systemic metabolism	Favorable safety profile; limited pediatric efficacy data; lack of standardized pediatric dosing regimens	Not recommended as standalone; possible adjunct in selected cases	Pediatric RCTs; dose–response relationship; comparison with cranberry	(18, 29, 30)
Micronutrients (Vitamin A, C and D)	25(OH)D, retinoids, ascorbic acid	Induction of antimicrobial peptides (e.g., cathelicidin LL-37); enhancement of epithelial barrier integrity; potential immunomodulatory effects on innate immunity (including TLR-related pathways); antioxidant activity and modulation of urinary microenvironment	Low (predominantly observational; limited interventional data)	Variable; absorption and effect dependent on baseline nutritional status and deficiency; systemic distribution with tissue-specific activity	Effect primarily observed in deficient populations; age-related variability in vitamin metabolism; potential interaction with immune maturation and microbiome composition	Selective use in children with documented or suspected deficiencies; targeted supplementation rather than routine empirical prophylaxis	Well-designed pediatric RCTs; dose optimization; role within multimodal strategies; identification of deficiency-driven responder subgroups	(14, 26–28)

Evidence levels reflect overall study quality and consistency across available literature. Mechanistic pathways are derived from experimental and translational data and should be interpreted in the context of clinical heterogeneity. The table is based on evidence derived from key mechanistic, clinical, and meta-analytic studies (1, 5, 7–10, 12–15, 18, 21, 22, 27, 29, 30).

Source: Author’s original work.

PACs proanthocyanidins; DMAC, 4-dimethylaminocinnamaldehyde assay; SCFAs short-chain fatty acids; TLR toll-like receptor; rUTIsrecurrent urinary tract infections; RCTs randomized controlled trials; LL-37 cathelicidin antimicrobial peptide.

### 6.2 Why single interventions may be insufficient

An important consideration in the prevention of rUTIs is the multifactorial nature of disease pathophysiology, involving bacterial adhesion, host immune responses, epithelial integrity, and microbiome dynamics (16, 35), suggesting that single-target interventions may be unlikely to provide sustained protection when used in isolation.

Cranberry-derived PACs primarily interfere with bacterial adhesion, whereas probiotics aim to restore microbial balance, and micronutrients support host immune and epithelial function, representing complementary rather than overlapping mechanisms, which may explain the modest and inconsistent effects observed with single interventions (14, 21).

This concept is particularly relevant in pediatric populations, where recurrence risk may be influenced by additional factors, including bowel dysfunction, microbiome maturation, nutritional status, and anatomical heterogeneity, further reinforcing the biological plausibility that effective prevention may require simultaneous targeting of multiple pathways. Although this hypothesis has not yet been adequately tested in pediatric randomized trials, it provides a coherent framework for interpreting the limitations of single-intervention approaches and supports further investigation of multimodal strategies.

### 6.3 Toward an integrated prevention model

Despite increasing research activity in this field, important evidence gaps remain, as many studies are limited by small sample sizes, heterogeneity in design, and lack of standardization in intervention formulations and dosing, with pediatric-specific data remaining particularly scarce for several interventions, including probiotics and D-mannose, and long-term outcomes rarely assessed (18, 21, 30).

In addition, few studies directly compare non-antibiotic strategies with each other or with antibiotic prophylaxis, limiting the ability to define optimal preventive approaches (16), while variability in outcome definitions, particularly regarding recurrence and microbiological endpoints, further complicates interpretation and comparability across studies.

Beyond methodological considerations, clinical implementation is influenced by real-world patterns of use, with available clinical data and observational studies suggesting that cranberry-based interventions are most frequently implemented as first-line non-antibiotic strategies, particularly in otherwise healthy children and in those without significant urological abnormalities (7, 20). Importantly, rUTIs in pediatric populations are frequently associated with functional urinary disorders, including bladder bowel dysfunction, dysfunctional voiding, constipation, vesicoureteral reflux, and neurogenic lower urinary tract dysfunction, all of which may contribute to urinary stasis, impaired bladder emptying, altered urinary ecology, and increased susceptibility to recurrent infection (16).

Among these factors, bowel dysfunction and constipation appear particularly relevant, given their recognized association with both urinary symptoms and alterations in gut microbial composition, further supporting the concept of interconnected gut–bladder axis mechanisms in pediatric rUTIs (16, 21). In addition, children requiring clean intermittent catheterization represent a clinically distinct population characterized by chronic bacterial exposure and increased recurrence risk (31).

Probiotics may be preferentially considered in children with coexisting bowel dysfunction, reflecting the recognized association between constipation and rUTIs (21, 22). Micronutrient supplementation appears most relevant in the presence of documented or suspected deficiencies (14, 27). D-mannose currently has a limited role in pediatric practice due to insufficient supporting evidence (18, 30).

**Figure 2:**
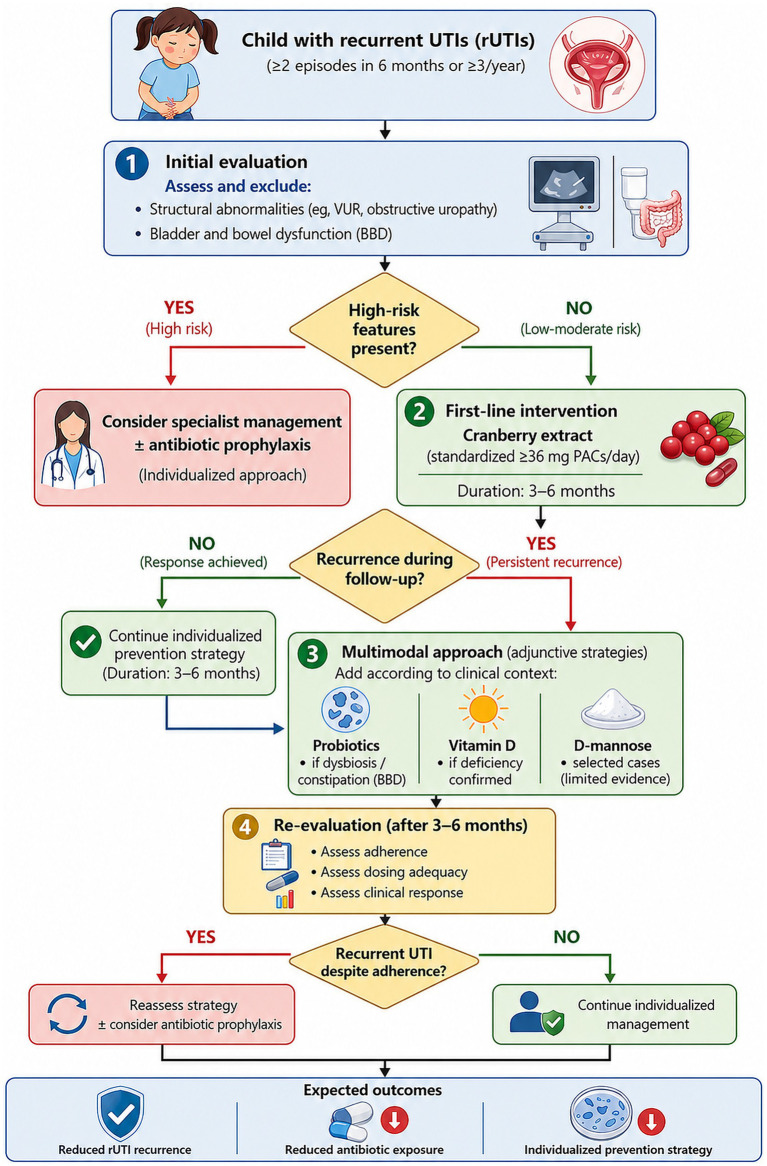
Proposed stepwise clinical algorithm for non-antibiotic prevention of recurrent urinary tract infections in children. The algorithm integrates current evidence and clinical practice patterns to support individualized prevention strategies in children with rUTIs. Proposed interventions include risk stratification, functional urinary evaluation, cranberry-based first-line prevention, adjunctive multimodal approaches and reassessment according to clinical response. The framework should be interpreted as hypothesis-generating and not as a substitute for individualized clinical judgment. Source: Author’s original work. rUTIs, recurrent urinary tract infections; PACs, proanthocyanidins; BBD, bladder and bowel dysfunction; VUR, vesicoureteral reflux; AMR, antimicrobial resistance. Figure was generated using ChatGPT and the prompt used can be found in Supplementary Table 1.

Collectively, these observations support the concept of an integrated and individualized, multimodal prevention model that combines nutritional interventions with individualized clinical assessment. Within this framework, non-antibiotic preventive strategies should be interpreted as adjuncts to comprehensive pediatric management rather than isolated uniform preventive interventions, with preventive approaches ideally adapted according to underlying functional abnormalities, recurrence phenotype, microbiome-related factors, and patient-specific clinical characteristics (16, 31). Several proposed microbiome-related and immunomodulatory interactions within this framework are currently supported primarily by mechanistic and translational evidence, while high-quality pediatric interventional data remain limited. A proposed clinical algorithm integrating risk stratification and non-antibiotic preventive approaches is presented in Figure 2.

### 6.4 Principal findings

The present synthesis highlights a clear gradient in the strength of evidence across non-antibiotic strategies for the prevention of rUTIs in children, with cranberry products demonstrating the most consistent and clinically relevant signal, supported by randomized trials and high-quality meta-analyses indicating a reduction in recurrence risk (7, 20). In contrast, probiotics, micronutrients, and D-mannose are characterized by more heterogeneous and frequently inconclusive data, with limited and inconsistent evidence supporting their effectiveness as standalone interventions (18, 21), likely reflecting both methodological heterogeneity and the multifactorial biology of pediatric rUTIs (16).

### 6.5 Mechanistic integration

A key observation emerging from this review is that the pathophysiology of rUTIs extends beyond a single mechanistic pathway, as bacterial adhesion, microbial virulence, host immune responses, and microbiome dynamics interact in a complex and interdependent manner (10, 35), reinforcing the limitations of single-target interventions. Within this framework, the observed clinical benefits of cranberry products, the variable effects of probiotics, and the context-dependent role of micronutrients appear biologically interconnected rather than contradictory.

Importantly, available clinical data and observational studies further support this interpretation, indicating that the combined or sequential use of non-antibiotic strategies may provide additive benefits, particularly when aligned with individual patient characteristics (16, 36). However, several proposed microbiome-mediated and immunomodulatory interactions underlying these approaches remain supported primarily by mechanistic and translational evidence rather than by clinically validated pediatric outcome data. Consequently, an integrated, multi-target approach may better reflect the biology of recurrent infections than isolated interventions.

### 6.6 Clinical implications

From a clinical perspective, these findings support a shift toward individualized, non-antibiotic prevention strategies in selected pediatric populations, as antibiotic prophylaxis, although still applicable in specific high-risk settings, is increasingly challenged by concerns related to AMR and modest clinical benefit (5, 6). Contemporary guideline frameworks similarly emphasize risk-adapted management and careful patient selection (32, 37).

In this context, cranberry products may be considered a reasonable first-line non-antibiotic option in otherwise healthy children with rUTIs. In contrast, probiotics and micronutrient-based approaches may have a complementary role, particularly in the presence of contributing factors such as microbiome imbalance or nutritional deficiencies. In addition, the identification of clinical predictors of recurrence, including anatomical and microbiological factors, may further support stratified prevention strategies (38). Overall, such an approach may help reduce unnecessary antibiotic exposure while maintaining effective prevention of recurrence.

### 6.7 Limitations

Several limitations should be considered when interpreting the available evidence, as the literature is characterized by substantial heterogeneity in study design, intervention formulations, dosing regimens, and outcome definitions, limiting comparability across studies and complicating the interpretation of pooled estimates (7, 8). In addition, pediatric-specific randomized controlled trials remain relatively limited for several interventions, particularly probiotics and D-mannose, resulting in uncertainty regarding their true clinical effectiveness (18, 21).

Furthermore, available clinical data derived from real-world practice, while valuable, are inherently subject to potential biases, including the absence of control groups and variability in adherence and co-interventions. These limitations highlight the gap between clinical practice and high-quality evidence and reinforce the need for cautious interpretation.

### 6.8 Future directions

Future research should prioritize well-designed, adequately powered randomized controlled trials in pediatric populations, particularly those evaluating combination strategies targeting multiple pathogenic pathways, which may provide more clinically meaningful insights than single-intervention approaches (14, 35).

Standardization of intervention characteristics, including dosing, formulation and duration, will be essential to improve reproducibility and comparability across studies (7, 8), while methodological inconsistencies in PAC quantification, particularly differences between assays such as 4-dimethylaminocinnamaldehyde assay (DMAC) and other analytical approaches, continue to limit dose–response interpretation and cross-study comparisons (8, 19).

Pediatric populations introduce additional complexity due to heterogeneity in age, microbiome composition, urinary tract anatomy, and coexisting functional disorders such as bladder and bowel dysfunction, with microbiome-dependent mechanisms likely contributing to inter-individual variability in treatment response.

Future research should integrate microbiome profiling, biomarker-driven endpoints, and clinical risk stratification tools to better identify responders and optimize individualized prevention strategies, including the incorporation of biomarkers such as urinary PAC metabolites, antimicrobial peptides (e.g., cathelicidin LL-37), and markers of epithelial integrity and inflammation (38). However, many of these proposed biomarker-driven and microbiome-oriented approaches currently remain exploratory and require validation in adequately powered pediatric clinical studies before routine clinical implementation can be considered. Such approaches may help bridge the gap between mechanistic plausibility and clinically meaningful outcomes in pediatric populations.

Finally, given the global challenge of AMR, further investigation into effective non-antibiotic alternatives remains a priority, as strategies that reduce reliance on antibiotics while maintaining clinical efficacy may play an increasingly important role in addressing the long-term burden of rUTIs in children (6, 39).

## 7 A proposed framework for non-antibiotic prevention

Given the multifactorial nature of rUTIs and the recognized limitations of single-intervention approaches, an integrated preventive strategy may be more appropriate in clinical practice, as combinations of non-antibiotic measures targeting complementary biological pathways are likely to provide a more effective and physiologically coherent approach than isolated interventions. Based on the integration of current evidence and clinical practice patterns, a stepwise and individualized management framework can be proposed to support clinical decision-making, while explicitly accounting for the heterogeneity of pediatric populations and variability in underlying risk factors.

At the initial level, children with rUTIs should undergo structured risk stratification. This evaluation should include assessment for bladder bowel dysfunction, constipation, dysfunctional voiding, and underlying urinary tract abnormalities, age, recurrence burden, and prior antibiotic exposure, as these factors may substantially influence recurrence risk and response to preventive interventions. Within this context, and particularly in otherwise healthy children without high-risk features, first-line preventive strategies may include cranberry-derived products administered at adequate PAC doses, given their favourable safety profile and the most consistent evidence among currently available non-antibiotic interventions (7, 16, 20). Cranberry-based preventive strategies appear most applicable in otherwise healthy children with recurrent uncomplicated UTIs, particularly when administered for sustained periods of approximately three to 6 months using formulations providing adequate PAC exposure.

In cases of persistent recurrence or in children with additional risk factors, including bowel dysfunction, microbiome-associated factors, or underlying urinary tract abnormalities, a multimodal approach may be considered, incorporating probiotics and selected micronutrients alongside cranberry supplementation. Such combinations may theoretically address multiple dimensions of UTI pathophysiology, including bacterial adhesion, microbiome composition, and host immune response, although it should be emphasized that current evidence supporting combined use remains limited and largely indirect (14, 21).

D-mannose may be considered in selected cases; however, its role remains less well established, as available data are heterogeneous and do not consistently support its use as a standalone preventive strategy, particularly in pediatric populations (18, 30).

Importantly, this proposed framework should be interpreted as hypothesis-generating rather than evidence-definitive, given the lack of high-quality pediatric studies evaluating combined non-antibiotic strategies. Furthermore, several proposed microbiome-mediated and immunomodulatory interactions underlying this framework are currently supported primarily by mechanistic and translational evidence rather than by clinically validated pediatric outcome data. Clinical decision-making should therefore remain individualized, considering patient characteristics, treatment tolerability, caregiver preferences. Such strategies should be integrated within routine pediatric evaluation, including assessment of recurrence burden, bowel and bladder dysfunction, prior antibiotic exposure, nutritional status, and underlying urological abnormalities.

Within the broader context of antimicrobial stewardship, such an approach may contribute to reducing cumulative antibiotic exposure while maintaining clinical safety, although this potential benefit requires confirmation in prospective pediatric studies (6, 39).

## 8 Conclusion

The prevention of rUTIs in children remains a complex and multifactorial challenge, in which no single intervention appears universally effective. The synthesis of current evidence, supported by mechanistic insights and clinical experience, suggests that non-antibiotic strategies are most likely to be effective when aligned with infection biology rather than applied in isolation.

Among available options, cranberry products emerge as the most consistently supported intervention, particularly when administered in formulations providing approximately 36 mg of PACs daily. Within a broader preventive framework, a combined strategy incorporating cranberry, selected probiotic strains and targeted micronutrient support may offer complementary benefits by addressing bacterial adhesion, microbiome dynamics and host immune function. However, several proposed interactions underlying these multimodal approaches remain supported predominantly by mechanistic, experimental, or translational evidence, while pediatric clinical validation remains comparatively limited. When applied over a defined period, typically three to 6 months, such an approach may reduce recurrence burden in selected pediatric patients.

Importantly, the potential benefit of this multimodal strategy should be interpreted as context-dependent rather than universally applicable. Its effectiveness is likely to vary according to patient characteristics, including age, recurrence pattern, underlying urological conditions and baseline nutritional status. In this setting, individualized implementation remains central, with careful patient selection.

From a broader perspective, these findings support a shift toward integrative non-antibiotic prevention models that aim to reduce reliance on long-term antimicrobial prophylaxis while preserving clinical effectiveness. Although current data are encouraging, they remain insufficient to support definitive recommendations regarding combined interventions. Further well-designed pediatric studies are required to confirm efficacy, define optimal dosing strategies and clarify the role of each component within combination regimens.

Ultimately, advancing the prevention of rUTIs in children will depend on integrating mechanistic understanding with high-quality clinical evidence, enabling the development of targeted, safe and sustainable strategies that reflect the complexity of this condition.
