# Supplementary material for: Nutritional bioactives for preventing recurrent urinary tract infections in children: microbiome-mediated mechanisms and clinical implications
Source: Front Nutr. 2026 Jun 10;13:1855301. doi: 10.3389/fnut.2026.1855301 (PMC13296944; doi:10.3389/fnut.2026.1855301)
Supplement: Supplementary file 1 [file Table_1.docx]

Supplementary materials with prompts for Figure 1 and Figure 2

- Name: ChatGPT
- Developer: OpenAI
- Model: GPT-5
- Version: ChatGPT web interface (GPT-5)
- Source: OpenAI

Figure 1 Prompt
"Create a scientific infographic illustrating how cranberry-derived proanthocyanidins, probiotics, vitamins A, C and D, and D-mannose may contribute to the prevention of recurrent urinary tract infections in children through microbiome-mediated mechanisms, modulation of host immune responses, effects on the urothelium, inhibition of bacterial adhesion, and reduction of recurrent infections, antibiotic exposure, and antimicrobial resistance pressure."

Figure 2 Prompt
"Create a clinical flowchart illustrating a stepwise non-antibiotic prevention strategy for recurrent urinary tract infections in children, including risk stratification, assessment of urinary and bowel dysfunction, cranberry-based prevention, escalation to probiotics, vitamin D, selected micronutrients, and D-mannose when appropriate, followed by reassessment and individualized management."
The initial figure concepts generated with ChatGPT were subsequently reviewed and substantially refined by the author using image-editing software. The final published figures were manually edited, scientifically validated, and approved by the author.
